# 

**Published:** 1894-11

**Authors:** 


### BOOKS RECEIVED.

Diseases of the Chest, Throat and Nasal Cavities. Including Physical Diagnosis and Diseases of the Lungs, Heart and Aorta, Laryngology and Diseases of the Pharynx, Larynx, Nose, Thyroid Gland and Esophagus. Third edition, revised. With 240 illustrations, 8vo, 718 pages. By E. Fletcher Ingals, A. M., M. D., Professor of Laryngology and Practice of Medicine, Rush Medical College; Professor of Diseases of the Throat and Chest, Northwestern University Woman's Medical School, etc., etc. Price, muslin, $5.00. New York: William Wood & Company. 1894.

Transactions of the American Surgical Association, Volume the Twelfth. Edited by De Forest Willard, M. D., Recorder of the Association. Philadelphia: Printed for the Association and for sale by William J. Dornan. 1894.

Text-Book of Anatomy and Physiology for Nurses. Compiled by Diana Clifford Kimber, Graduate of Bellevue Training School; Assistant Superintendent, New York City Training School, Blackwell's Island, N. Y. Octavo pp. xvi.—245. Price, $2.50. New York and London: Macmillan and Co. 1894.

The Sympathetic Nerve. Drawn from a dissection prepared by Byron Robinson, M. D. Chicago: 1894.

A Manual of Hygiene. By Mary Taylor Bissell, Professor of Hygiene in the Woman's Medical College of the New York Infirmary for Women and Children. Octavo, cloth, $2.00. New York: The Baker & Taylor Co., 5 and 7 East Sixteenth street. 1894.

Eighteenth year. Book of the New York State Reformatory, Elmira, N Y. Containing the annual report of the board of managers for the year ending September 30, 1893. Transmitted to the State Legislature, January, 1894.

Travaux D'Electrothérapie Gynécologique Archives Lemestrielles D'Electrothérapie Gynécologique Fondées et Publiées par Le Dr. G. Apostoli, Vice-Président de la société Francaise D'Electrothérapie, etc. Volume Fascicules 1 et 11. Paris: 1894.

Syllabus of Lectures on Human Embryology: An Introduction to the Study of Obstetrics and Gynecology. For Medical Students and Practitioners. With a Glossary of Embryological Terms. By Walter Porter Manton, M. D., Professor of Clinical Gynecology and Lecturer on Obstetrics in the Detroit College of Medicine; Fellow of the Royal Microscopical Society, of the British Zoological Society, American Microscopical Society, etc., etc. Illustrated with Seventy (70) Out'ine Drawings and Photo-Engravings. Duodecimo, cloth, 126 pages, interleaved for adding notes and other illustrations, $1.25 net. Philadelphia: The F. A. Davis Co., Publishers, 1914 and 1916 Cherry Street. 1894.

